# Assessment of Brain Morphological Abnormalities and Neurodevelopmental Risk Copy Number Variants in Individuals from the UK Biobank

**DOI:** 10.3390/ijms26157062

**Published:** 2025-07-22

**Authors:** Sara Azidane, Sandra Eizaguerri, Xavier Gallego, Lynn Durham, Emre Guney, Laura Pérez-Cano

**Affiliations:** 1 STALICLA Discovery and Data Science Unit, World Trade Center, Moll de Barcelona, Edif Este, 08039 Barcelona, Spain; sara.azidane@stalicla.com (S.A.); 01eizaguerrisandra@gmail.com (S.E.); xavier.gallego@stalicla.com (X.G.); lynn.durham@stalicla.com (L.D.); emre.guney@stalicla.com (E.G.); 2 Institut de Biotecnologia i de Biomedicina, Universitat Autònoma de Barcelona, Bellaterra, 08193 Barcelona, Spain

**Keywords:** brain morphology, copy number variants, NDD symptoms, brain development, UK biobank, psychiatric cohort, NDD cohort

## Abstract

Brain morphological abnormalities are common in patients with neurodevelopmental disorders (NDDs) and other neuropsychiatric disorders, often reflecting abnormal brain development and function. Genetic studies have found common genetic factors in NDDs and other neuropsychiatric disorders, although the etiology of brain structural changes in these disorders remains poorly understood. In this study, we analyzed magnetic resonance imaging (MRI) and genetic data from more than 30K individuals from the UK Biobank to evaluate whether NDD-risk copy number variants (CNVs) are also associated with neuroanatomical changes in both patients and neurotypical individuals. We found that the size differences in brain regions such as corpus callosum and cerebellum were associated with the deletions of specific areas of the human genome, and that specific neuroanatomical changes confer a risk of neuropsychiatric disorders. Furthermore, we observed that gene sets located in these genomic regions were enriched for pathways crucial for brain development and for phenotypes commonly observed in patients with NDDs. These findings highlight the link between CNVs, brain structure abnormalities, and the shared pathophysiology of NDDs and other neuropsychiatric disorders, providing new insights into the underlying mechanisms of these disorders and the identification of potential biomarkers for better diagnosis.

## 1. Introduction

Brain morphological abnormalities—structural anomalies in brain’s anatomy—are often associated with neurodevelopmental and psychiatric disorders. Different conditions, such as schizophrenia, autism spectrum disorder, depression, or substance use disorder, among others, can manifest as specific patterns of brain structural abnormalities. These variations in morphology can include changes in brain size, shape, cortical thickness, folding patterns, and volumes of subcortical structures such as the amygdala, hippocampus, corpus callosum, or cerebellum [1,2,3,4].

For example, the abnormality of cortex thickness has been reported in patients with autism spectrum disorder and is involved in social, emotion, and language processing [5,6,7,8]. Also, adults with major depressive disorder have shown a pattern of gray matter thinning in areas key to emotional regulation and cognitive/sensory processing [9]. Similarly, patients with attention-deficit/hyperactivity disorder (ADHD) have consistently showed global decreased cortical thickness, as well as thinning of specific regions involved in attention and executive function, such as the prefrontal cortex or the anterior cingulate area [10,11].

Subcortical structures also show a variety of abnormalities in patients with neurodevelopmental disorders (NDDs) and other neuropsychiatric disorders. For instance, increased amygdala volumes are commonly reported in patients with autism spectrum disorder (ASD), corresponding with anxiety and fear behaviors especially in the stages of childhood and adolescence [12,13]. In ADHD, patients consistently display a decreased amygdala volume, which is thought to mirror impulsive patterns characteristic of the disorder [14]. In the same way, substance use disorders involving alcohol or cannabis have also been seen in association with amygdala volume reductions [2], which appear to predate substance use and may reflect a pre-existing risk factor for the development of substance use disorders, rather than solely a consequence of substance-induced neurotoxicity [15]. Other affected traits in patients with NDDs and neuropsychiatric disorders can include the size of the ventricles [16], the volume of the cerebellum [17], the total brain volume [18], or the surface area of the brain [19], in addition to other brain measures [20].

In recent years, the genetic etiology of these morphological abnormalities in patients with NDDs has begun to be explored, and it has been observed that copy number variants (CNVs) play an important role [21]. CNVs are large nucleotide sequences that are deleted or duplicated and can span from several kilobases (kb) to megabases (Mb). These types of structural variants are known to be strongly associated with the risk of developing neurodevelopmental disorders such as autism, ADHD, and schizophrenia [22,23], among others. Evidence increasingly suggests that the pathogenicity of these variants is often driven by dosage sensitivity mechanisms—whereby deletions or duplications disrupt the function of genes that are particularly sensitive to changes in copy number [24,25]. Nevertheless, the mechanisms and phenotypes underlying these genomic changes are not yet fully understood. This is due to the rarity of these variants in the population and properly analyzing them requires large cohorts of patients. Also, these variants are large and may affect many genes at the same time, making it hard to pinpoint which gene or genes are responsible for the manifestation of the disorders or the abnormal trait [24].

Genomic disorders, i.e., conditions resulting from the deletions or duplications of the human genome, such as 22q11.2 syndrome, 15q11.2 syndrome, 1q21.1 syndrome, and 16p11.2 syndrome, among others, have earlier highlighted the connection between gene dosage and general brain morphology measures. Recent technological advances in sequencing and imaging, such as the development of whole-genome sequencing (WGS) and mass sequencing in large-scale biobanks, allow analyzing the impact of CNVs on the brain structure across different global measures. CNVs can have global effects such as changes in total intracranial volume (TIV), surface area (SA), or average cortical thickness (CT). For example, 16p11.2 deletions and 22q11.2 duplications are known to increase TIV [26,27], while 16p11.2 duplications and 22q11.2 deletions have been associated with lower TIV [27,28]. Also, variants in carriers with deletions or duplications in 1q21.1 have reported an effect on TIV, as evidenced by the high prevalence of microcephaly and macrocephaly, respectively [29].

Similarly, correlations have been observed between gene dosage and subcortical brain morphological measures. For example, an inverse relationship has been observed between gene dosage and the volume of certain brain regions: deletions at 16p11.2, which reduce gene dosage, are associated with the increased volume of subcortical structures such as the caudate, pallidum, and putamen, whereas duplications at the same locus, which increase gene dosage, lead to a decreased volume in these same areas [21]. Likewise, the caudate and hippocampus show reduced volume with an increase in the copy number at the 1q21.1 region [30].

While the link between CNVs and brain morphology has been explored for regions recurrently reported to be associated with NDDs (e.g., 1q21.1, 15q11.2, 16p11.2, 16p13.11, and 22q11.2) [31,32], the effect of other NDD-risk regions in brain morphology have been less studied, possibly due to their lower frequency in cases and the lack of large-scale cohorts that allow for a powered statistical analysis. For example, this is the case for regions such as 6p25, 8p23, and 4p16.3, where fewer patients are reported to be CNV carriers in these regions as compared to other recurrent CNVs. Moreover, to date, neither the frequencies of certain risk variants in the general population nor the penetrance of these CNVs are known with certainty, both in terms of diagnosis and brain structure abnormalities. Studies with large-scale data allow for more accurate and less inflated estimates of these parameters compared to clinical cohorts. Furthermore, identifying these estimates would not only assist in understanding the pathogenicity of these variants, but also enable improved genetic counseling for families and patients.

In our recent meta-analysis of CNV data from 11,614 affected individuals with NDDs and 4031 control individuals from the SFARI database (https://gene.sfari.org) [25], we identified 41 NDD-risk CNV loci, including 24 novel regions. Furthermore, in that analysis [25], we observed several associations between some of the genes harboring these NDD-risk CNVs and those associated with brain morphological abnormalities, such as hypoplasia of the corpus callosum or microcephaly. In this work, we systematically investigate specific brain alterations associated with this previously defined set of NDD-risk CNVs using the UK Biobank (UKBB) data. Also, considering the genetic overlap between neuropsychiatric disorders and NDDs—evidenced by the high prevalences of neuropsychiatric disorders in families of individuals with NDDs—we aimed to explore the association between NDD-risk CNVs and brain morphological abnormalities in both patients with NDDs and neuropsychiatric disorders.

We employed the extensive genetic, clinical, and brain imaging data from hundreds of thousands of individuals in the UKBB for studying rare variant–morphology associations. By analyzing brain morphology associations extracted from imaging data combined with the genetic data, we aim to improve our understanding of the pathophysiology underlying the brain morphological manifestations of various NDD-risk CNVs that have not been fully studied yet in patients with NDDs and psychiatric disorders.

## 2. Results

### 2.1. Association Between Abnormal Brain Morphological Traits and Neuropsychiatric Disorders

Brain imaging (MRI T1) data were analyzed from 31,929 participants from the UKBB, including 9798 participants with a psychiatric diagnosis and 22,131 control participants. We defined abnormal brain morphological traits (AMTs) in the dataset as those brain morphological features with values outside a ±2 Z-score threshold (see Section 4). Then, we hypothesized that some of these AMTs might be related to pathological conditions associated with NDDs and other psychiatric disorders.

**Figure 1:**
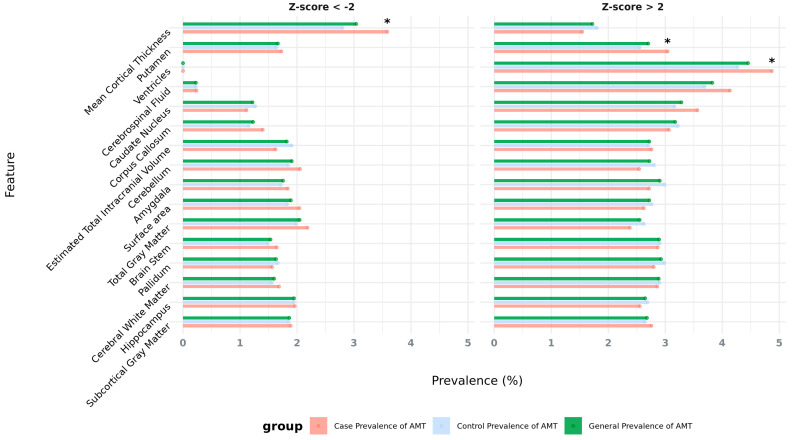
The prevalence of extreme aberrant morphological traits (AMTs) in the UKBB population, color coded by case (red), control, (blue) and general, i.e., both cases and controls are combined (green). Asterisks indicate significant associations.

First, we examined the prevalence of these AMTs across multiple brain regions to quantify the burden of structural abnormalities in the population. We saw that the general prevalence of extreme values (or abnormalities, combining both cases and controls) ranges from 0% to 4.5% across different brain areas, with the most prevalent features being enlarged ventricles (4.5% with Z-score > 2), increased cerebrospinal fluid (3.9% with Z-score > 2), enlarged caudate nucleus (3.3% with Z-score > 2), and enlarged corpus callosum (3.2% with Z-score > 2). In this sense, we can observe that, for most brain areas, abnormalities are more prevalent in the Z-score > 2 direction compared to the Z-score < −2 direction. In particular, the average prevalence of enlargement abnormalities (Z-score > 2) is 3%, while that of reduction abnormalities (Z-score < −2) is 1.6%, suggesting that the distributions may be skewed towards enlargement rather than reduction in size or volume of the assessed brain morphological features or image-derived phenotypes (IDPs) (Figure 1, Supplementary Table S2). A Wilcoxon test further confirmed this trend, showing that Z-score > 2 has a significantly higher mean prevalence than Z-score < −2 (V = 491, *p*-value = 5.31 × 10^−9^).

Our analysis also aimed to determine whether these abnormalities are more common in NDD and psychiatric cases compared to neurotypical controls. While no significant association was identified when considering all abnormal morphological traits in general (Welch Two-Sample *t*-test: *p*-value = 0.48; 95% CI −9.6 × 10^−3^ to 4.7 × 10^−3^; mean prevalence of abnormalities in cases = 2.3%; mean prevalence of abnormalities in controls = 2.3%), this analysis revealed that 18 features out of 31 (58.1% of the assessed IDPs) had a higher prevalence in cases vs. controls. This includes several AMTs that are statistically significantly enriched in individuals with a psychiatric diagnosis (Supplementary Table S2).

For example, results indicate significant associations between an increased putamen volume (Z-score > 2) and NDDs/psychiatric disorders (OR = 1.20, *p*-value = 1.34 × 10^−2^, prevalence _cases_ = 3.1%, prevalence _controls_ = 2.8%). Similarly, increased ventricle volumes (Z-score > 2) are also significantly associated with case status (OR = 1.15, *p*-value = 1.71 × 10^−2^, prevalence _cases_ = 4.9%, prevalence _controls_ = 4.3%). The most significant finding is related to the enrichment of decreased mean cortical thickness in cases (Z-score < −2) (OR = 1.29, *p*-value = 2 × 10^−4^, prevalence _cases_ = 3.6%, prevalence _controls_ = 2.8%).

### 2.2. Subjects with Deletions or Duplications in NDD-Risk Regions Are Enriched for Aberrant Morphological Brain Traits

**Figure 2:**
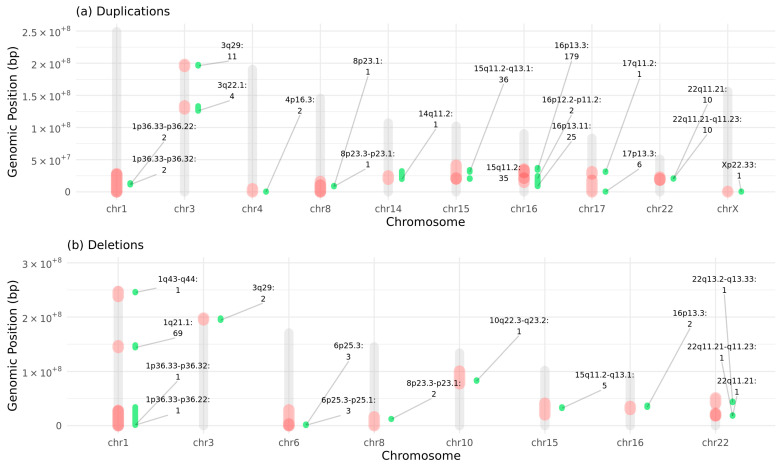
Genomic regions (in red) that entail a risk for NDDs and brain abnormalities, overlapped by rare deletions (**a**) and rare duplications (**b**) of the UKBB cohort. The variants that overlap these regions are depicted in green, parallel to the red area that marks known NDD-risk region of the chromosome, along with the number of patients reported for the variant in that specific region.

CNVs in NDD-risk regions have been linked to structural brain alterations. Here, we identified a set of 228 high-confidence CNVs in 371 participants (i.e., both cases and controls) of our cohort following stringent filtering, revealing recurrent deletions and duplications in NDD-risk loci established in our previous analysis [25]. Figure 2 presents the distribution of these qualifying CNVs, i.e., both deletions and duplications, across the chromosome regions, accompanied by the number of patients (both cases or controls) carrying a variant in a specific NDD-risk region.

A burden analysis in the UK Biobank population revealed no significant enrichment of NDD-risk genetic variants in psychiatric patients compared to controls when assessed region by region (Supplementary Table S3). To complement this, we performed a joint analysis considering the overall burden of all NDD-risk CNVs combined. Similarly, the joint burden analysis of all CNVs across cases and controls did not reveal a significant difference (Fisher’s exact test, *p* = 0.864, OR = 1.02, 95% CI: 0.81–1.28). This failure to identify well-known previously reported regions enriched in cases may be attributed to limitations on the analysis. Investigating the statistical power of an unmatched case–control study design, we computed the metrics using classical epidemiological methods (see Appendix A.1), and it yielded a beta value of 5.41 × 10^−2^, denoting an underpowering of the study in this specific analysis.

We then evaluated the enrichment of neuroanatomical malformations in individuals (regardless of affectation status, i.e., considering both cases and controls) carrying deletions or duplications in NDD-risk regions compared to non-carriers (Supplementary Table S4). Despite the low beta value and how unpowered the analysis was, we still observed some interesting trend associations between NDD-risk CNV regions and AMTs with an FDR of <0.2. In particular, we observed that the deletion of 3q29 was associated with reduced corpus callosum volume (*p* = 1.46 × 10^−4^, OR = Inf, CI = 15.33-Inf, FDR = 0.14, that passes the corrected alpha M_eff_ multiple test cut-off), and the deletion of 8p23.3-p23.1 is associated with reduced cerebellum volume (*p* = 3.53 × 10^−4^, OR = Inf, CI = 9.79-Inf, FDR = 0.17).

We then assessed the prevalence of AMTs (Z-score < −2 or Z-score > 2) among UKBB NDD-risk CNV carriers, revealing substantial penetrance variability (Supplementary Table S4). For example, deletions on 1p36.33-p36.22 and 10q22.3-q23.2 (with one patient reported each) have shown an abnormally large cerebellum, reporting a 100% prevalence of cerebellum Z-score greater than 2 associated with those regions. On the other hand, the 8p23.3-p23.1 region was deleted in two patients that exhibited an abnormally small cerebellum. There are NDD-risk regions more frequently affected by CNVs in participants but showing less penetrance for AMTs. For instance, 18.2% of individuals carrying deletions in the 3q29 region present an abnormally thicker gray cortex, and 10.3% of individuals with deletions in 1q21.1 show abnormally enlarged ventricles.

Furthermore, NDD-risk CNVs often showed association with abnormalities in multiple brain regions at once, i.e., a single deletion or duplication could be associated with several different AMTs. In this sense, for some NDD-risk regions, multiple AMTs were consistently linked to the same variant, and in some carriers, these alterations co-occurred within the same individual. For example, some carriers with 1q21.1 deletions show significant size or volume deviations in the caudate nucleus, cerebellum, cerebral white matter, hippocampus, putamen, subcortical gray matter, and ventricles. Another example is 15q11.2 duplication, a known NDD-risk CNV that we observe causing structural abnormalities in several parts of the brain, particularly in the cerebral white matter, corpus callosum, and mean cortical thickness (Supplementary Table S4).

### 2.3. Genes in AMT-Associated CNV Regions Show HPO Enrichment for Brain Development and Hallmark NDD Traits

**Figure 3:**
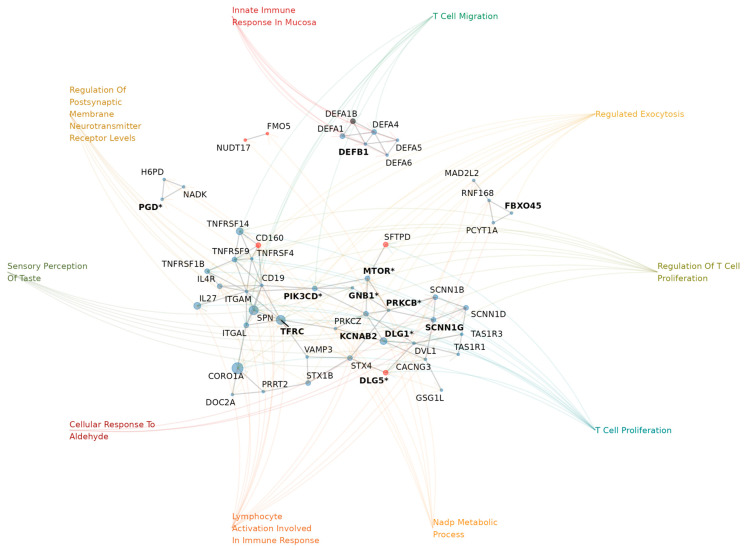
The protein–protein interaction (PPI) network of all protein-coding genes located in NDD-risk regions and significantly associated with AMT when affected by CNVs. The network highlights genes involved in enriched GO terms across the biological process (BP) domain. Node size reflects the number of GO pathways associated with each gene. Red nodes indicate genes within deletions, and blue nodes indicate genes within duplications. Genes in bold are dosage-sensitive, and genes marked with * are previously reported NDD-risk genes.

We then explored genes affected by CNVs within NDD-risk regions that show suggestive associations with AMTs (*p*-value < 0.05; from our previous analysis, Table S4). We found these genes to be enriched for Gene Ontology (GO) terms that are related to brain development and function. Specifically, we identified a total of 169 nominally significant enriched terms (*p*-value < 0.05 and FDR < 0.2), which represent a medium-confidence set, including a subset of 37 high-confidence terms that passed multiple test correction with an FDR of <0.05 (Figure 3).

Some of the medium-confidence biological processes that we identified are key pathways in brain development and function, such as neurotransmitter secretion and transport, the regulation of oligodendrocyte differentiation, protein localization to synapse/post-synapse, and signal release from synapse. Likewise, the regulation of myelination and its activation in immune response are significantly enriched, as well as pathways such as the G protein-coupled acetylcholine receptor signaling pathway or negative regulation of ERBB. Also, regarding the enriched cellular components in this set, we observed asymmetric synapse, postsynaptic density, and Set1C/COMPASS complex enrichment, among others (Supplementary Table S5).

Furthermore, we noted that high-confidence terms (i.e., surpassing multiple test correction) are also key pathways in brain formation and housekeeping. These include immunological mechanisms such as leukocyte, lymphocyte, or T-cell activation/migration, as well as other processes such as nicotinamide/pyridine metabolism or exocytosis. Of particular interest are the enriched cellular components observed in this gene set, such as the presynaptic and postsynaptic cytosolic machinery or the protein complex involved in cell adhesion, all three of which are crucial parts of the synaptic transmission. Similarly, several transporter activity functions are enriched, such as numerous sacarid membrane transportations as well as a tumor necrosis factor receptor (Supplementary Table S6).

In agreement with our previous study highlighting the role of dosage-sensitive genes in the pathogenicity of NDD-risk CNVs, we found that genes involved in the GO-enriched pathways were significantly enriched for dosage-sensitive genes (Fisher’s exact test, *p* = 1.8 × 10^−3^).

To further investigate this, we performed GO enrichment analysis restricted to the subset of dosage-sensitive genes within these regions. While this more stringent approach yielded fewer significant terms overall (40 nominally significant GO terms with *p* < 0.05, of which 9 survived FDR < 0.05 correction, Supplementary Table S7), the enriched pathways were also directly related to neurodevelopmental and synaptic functions—such as transcriptional regulation, neuronal differentiation, and synaptic signaling. Notably, high-confidence terms included components of the synapse (e.g., postsynaptic density, neuron-to-neuron synapse) and regulatory structures (e.g., nuclear speck, SCF ubiquitin ligase complex).

Finally, we identified Human Phenotype Ontology (HPO) terms to be enriched for both all genes and the subset of dosage-sensitive genes from NDD-risk regions that are associated with AMTs, with the former resulting in a larger set of enriched terms (Supplementary Table S8).

In particular, we identified 185 HPO terms enriched in association with dosage-sensitive genes from NDD-risk regions that are associated with AMTs (Supplementary Table S8), 67 of which had an FDR of < 0.05 (surpassing multiple test correction). We then performed a permutation test by conducting 200 HPO enrichment analyses using randomly selected gene sets. This approach was used to control for false positives and to evaluate the robustness of our findings. Notably, none of the empirical *p*-values derived from the permutations reached significance, indicating that the enrichments observed for the gene set derived from the AMT-associated CNV regions are unlikely to be due to random variation.

**Figure 4:**
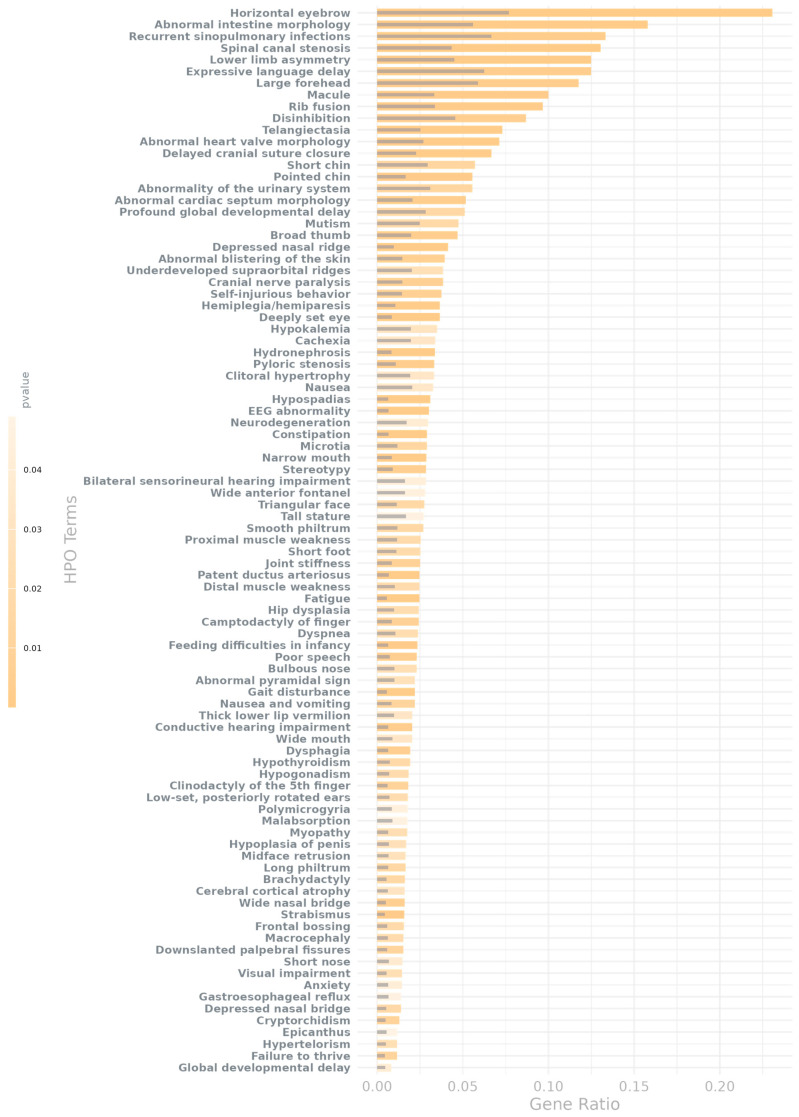
Observed vs. randomized gene overlap ratios for significant NDD-related HPO terms. Shown are HPO terms that are significantly enriched for qualifying dosage-sensitive genes—those located in NDD-risk regions suggestively associated with AMTs—and that passed multiple testing correction. For this analysis, NDD-related phenotypes are defined as terms with a reported prevalence in one or more neurodevelopmental disorders greater than 80 or that are commonly used as diagnostic criteria, as reported in Orphanet (the full list is in Supplementary Table S9). Colored bars show the observed overlap ratio for each phenotype, while gray bars indicate the mean overlap ratio from 200 randomized permutations, representing the level of gene overlap expected by chance.

In addition, many of these terms represent hallmark phenotypes of neurodevelopmental disorders or are directly linked to brain disorders (Figure 4). In fact, we found both the full set of nominally significant HPO terms (*p*-value < 0.05 and FDR value < 0.2) and the subset corrected for multiple testing (*p*-value < 0.05 and FDR value < 0.05) to be also statistically significantly enriched for known NDD-related phenotypes (i.e., phenotypes with an observed prevalence in NDDs higher than 80 or used as diagnostic criteria [33]; *p*-value = 3.58 × 10^−54^, OR = 13.33 for the full set; *p*-value = 2.28 × 10^−20^, OR = 12.70 for the corrected set; the list of known NDD-related phenotypes is in Supplementary Table S9). These phenotypes include polymicrogyria—the excessive formation of small convolutions—or macrocephaly as well as abnormalities in neuronal migration and morphology, such as abnormal upper motor neuron morphology, abnormal lower motor neuron morphology, and generalized cerebral and cortical atrophy or hypoplasia. In addition, we also found enrichment for cranial anomalies, such as a wide anterior fontanel, cranial nerve paralysis, and a delayed cranial suture closure, all of which are known symptoms of an alteration in the development of the skull and brain.

Besides brain structural abnormalities, NDDs are also characterized by cognitive and behavioral phenotypes that contribute significantly to the morbidity burden. Among them, genes of interest were also enriched for phenotype terms such as profound global developmental delay, stereotypes, hallucinations, apathy, anxiety, disinhibition, agitation, mutism, self-injurious behavior, and focal seizures, which are key indicators of neurodevelopmental impairment. In addition, we also found enrichment for terms related to motor and coordination difficulties, which are also commonly observed in NDD patients, e.g., gait disturbance, distal muscle weakness, hemiplegia or hemiparesis, and dysphagia, which may reflect cerebellar or cortical dysfunction. Interestingly, we also observed NDD-related EEG abnormalities enriched for this gene set, which further highlights alterations in neurophysiological activity associated with these risk regions.

In addition to core neurological and cognitive features, many individuals with NDDs present with systemic and comorbid conditions that, while not exclusive to these disorders, occur at a higher frequency. In this sense, we observed enrichment for terms like feeding difficulties in infancy, recurrent sinopulmonary infections, and failure to thrive. Other identified features include hypogonadism, cryptorchidism, hypospadias, renal cysts, hydronephrosis, joint stiffness, hip dysplasia, and abnormalities of the external ear, tongue, and facial morphology, reflecting the multisystemic nature of many NDDs.

## 3. Discussion

Our analysis of the UKBB database revealed AMTs that occur at rates of up to 4.5% in the UKBB population. Interestingly, we observed a consistent trend toward structural enlargement rather than volume reduction, a pattern that was statistically significant. As previously reported [34,35], this suggests that brain overgrowth may be a more prevalent form of morphological abnormality in the population than brain undergrowth.

**Figure 5:**
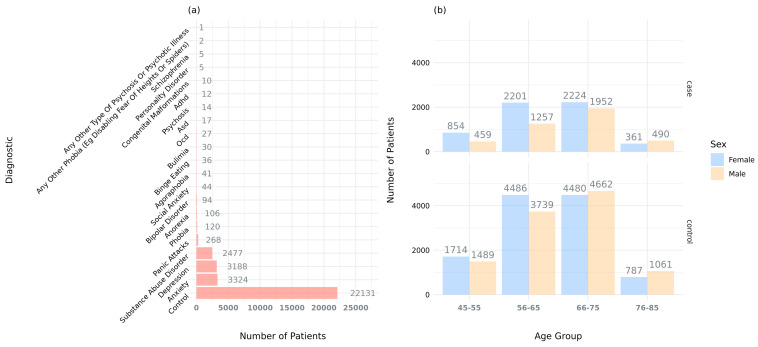
Demographics of study participants: (**a**) number of selected participants by diagnosis and (**b**) number of selected participants by age and sex, grouped by affectation status (e.g., case or control). Information also available in Supplementary Table S10.

When comparing individuals with NDDs to neurotypical controls, we found that 58.1% of the identified AMTs had a higher prevalence in cases. However, no significant overall association was observed, indicating that while most of the brain abnormalities were found to be more prevalent in patients, having any brain abnormality is not predictive enough to distinguish individuals with NDDs or other psychiatric disorders from neurotypical individuals in the UKBB. Nevertheless, it is worth mentioning that the UKBB is biased towards older participants (Figure 5), and it is therefore not representative of the general population. Despite this, specific traits such as increased putamen volume, enlarged ventricles, and reduced mean cortical thickness were significantly enriched in case individuals, suggesting that while structural variations in the brain can occur widely, certain patterns of abnormality may be more closely linked to neurodevelopmental or psychiatric disorders. In this sense, these three brain structures (i.e., putamen, ventricles, and cortex) have been previously reported in association with the etiology of this type of disorders.

For instance, cortical thinning is a well-documented symptom in a normal aging population, and it is associated with cognitive deterioration in the elderly [36]. Nevertheless, conditions such as schizophrenia [37], substance use disorder [38], and depression [39] show a more pronounced pattern of generalized cortical thickness reduction in the aging population. A thinner cortex is correlated with reduced neural connectivity [40] and altered neurotransmitter system [41], suggesting that the effects of reductions in cortex thickness associated with age may be exacerbated by dopamine-related disturbances in some neuropsychiatric disorders.

Also, we could identify a statistical association between enlarged putamen volume and patients with NDDs and other psychiatric disorders. This fact is particularly interesting, since the putamen—the structure involved in dopaminergic regulatory processes—is known to undergo volume loss with aging but an increase in its volume has been identified as a distinct marker of psychiatric disorders, but especially in dependence and substance use disorders [42]. This volume enlargement of the putamen can be attributed to the overexpression of dopaminergic neurons within the nigrostriatal pathway. This phenomenon may occur either due to an increased number of dopaminergic neurons or neuronal hypertrophy that results from a compensatory response to disrupted dopamine signaling characterized in psychiatric populations [42].

Ventricular enlargement is also a common feature of the aging brain [43] that serves as a marker of cognitive decline in aging, since it has been correlated with cognitive function and information processing [16]. In young brains, ventriculomegaly is also known to be associated with cognitive defects in some NDDs. It is, for example, one of the most consistent findings in schizophrenia [44] and developmental delay [45], but limited information in the literature exists associating this structure with the aging brain and the pathophysiology of neuropsychiatric disorders.

The penetrance of CNVs affecting brain structure in neurodevelopmental and psychiatric disorders is highly variable and often incomplete, as not all carriers of risk variants develop the full phenotype of the expected AMT. Although these variants are clearly enriched in patients and known to increase the risk for NDDs, the study of penetrance is challenging due to the complexity of these disorders, bias in cohorts, and the technical noise in brain imaging—particularly in subcortical structures—limiting the available data [46].

To date, several studies have attempted to use large databases such as the UKBB to report the true—or less biased—penetrance of these variants in AMTs [31,47], as this information helps quantify the risk severity for carriers and supports genetic counseling. Nevertheless, to our knowledge, no penetrance analysis has yet been conducted on such a large number of NDD-risk regions using WGS-derived variants, both in global measures such as CT, SA, TIV, ventricles, and brain stem volume as well as in the volumes of subcortical structures. In this sense, our results reflect the expected heterogeneity, showing a wide variability in carrier penetrance within the cohort, reinforcing the idea that these disorders are part of a continuum, and that high penetrance is not the norm for these conditions.

For example, in our analysis, only a few CNV–AMT associations showed 100% prevalence in carriers, such as 3q29 deletions, associated with corpus callosum hypoplasia. Nevertheless, although these results might suggest a complete penetrance, due to the rarity of the variants, sample size is too small to draw a definite conclusion on the complete penetrance of these CNVs. Other associations, with more patients reported by variant, display much lower prevalence, sometimes below 10%. For example, CNVs at 1q21.1 and 16p13.11 are associated with more modest penetrance values, indicating that not all carriers exhibit the associated neuroanatomical deviations, consistent with the incomplete penetrance that is more commonly observed. This also highlights the concept that, for certain variants, what is probably reflected at the phenotypic level is a symptomatic or mild clinical expression, explaining a “reduced” penetrance of the genotype, which does not reach the pathogenic status or clinical diagnosis threshold by itself. This intermediate penetrance characterizes precisely the regions 15q11.2 and 16p13.33, a fact that has not only been identified in our study but has also been reported in recent studies. When considering the penetrance of variants at the disorder level, we ratified the highly variable results expected in a non-clinical cohort. While some variants reported a higher OR and a higher burden in case carriers, e.g., 8p23.3, other variants affecting well-established NDD-risk regions showed a low case penetrance and even a negative odds ratio, which may suggest partial expressivity, as in the case of 17p13.3 or 1q21.

We did not observe an enrichment of known and previously reported NDD-risk variants in cases. Through a power calculation analysis, we established that this analysis was highly underpowered (beta = 5.5%, when at least beta > 80% is expected), which is explained by the rarity of these variants and a population that is considered to have a “healthy volunteer” selection bias [48]. However, we could observe that there is a trend of association between NDD-risk CNVs and specific AMTs.

Interestingly, these association trends between genetic variants and morphological abnormalities were observed independent of the diagnostic status. For instance, we identified the carriers of risk variants with atypical morphological features who have been classified as controls. This is particularly relevant for two reasons: first, it suggests that some individuals may carry risk genetic variants and present morphological alterations without ever fully developing the disease, i.e., without reaching the threshold necessary to obtain a diagnosis, further supporting the intermediate penetrance model discussed before. Secondly, it also raises the possibility that some cases have not yet been adequately diagnosed. However, given the limited power due to the lack of carriers in the UKBB cohort, multiple hypothesis testing correction is not possible, and these associations should be considered as potential trends pending validation.

Interestingly, some individuals with 1q21.1 deletions presented caudate morphological abnormalities, a brain structure that has been previously associated with complex motor mannerism and ritualistic repetitive behavior, which are the characteristics of stereotypical patients with these disorders [49,50]. In the same manner, some subjects with duplications at 16p13.11 had larger volumes of the amygdala, highlighting the involvement of this NDD-risk variant in emotional regulation, in agreement with previous reports showing that an enlarged amygdala is associated with higher rates of anxiety and phobias, which are characteristics of psychiatric disorders [51,52]. Also, some patients with this variant showed increased pallium sizes that are associated with the early signs of psychosis [53] and ASD [54]. Notably, the carriers of deletions at 1p36.33-p36.32/1p36.33-p36.22 also showed an increased amygdala size, in line with previous reports [55,56], suggesting a role in limbic system development.

Our results also show a link between brain morphology-altering NDD-risk variants and clinical phenotypes of NDDs including structural brain abnormalities. We identified key dysmorphic features through GO and HPO analyses, which correlate with the pathogenesis of neurodevelopmental abnormalities. The enriched brain morphological abnormalities, such as neuronal migration defects and atrophy/hypoplasia of the frontal lobes of the neocortex in the brain, are indicators of impaired brain development. In addition, enriched GO terms such as lymphocyte/leukocyte activation in immune response and abnormalities in the pyridine/NADP metabolism are also seen consistently in patients with NDDs [57]. Genes within brain morphology-altering NDD-risk variants participating in GO enriched pathways were also found to be enriched for dosage-sensitive genes. Notably, when focusing specifically on these dosage-sensitive genes, the GO enrichment remained strongly associated with neurodevelopmental processes, further underscoring their likely contribution to disease etiology.

Further, small volumes of cortex and subcortical structures such as the putamen, the caudate, and parts of the cerebellum suggest that CNVs located in the NDD-risk areas of the genome could be responsible for some degree of impairment in the control of movements, which we also see enriched in the HPO enrichment analysis. Motor–neurological impairments associated with the listed terms include gait disturbance, reduced tendon reflexes, hip dysplasia, joint stiffness, postural tremor, spinal cord compression, opisthotonus, progressive muscle weakness, hemiplegia/hemiparesis, cranial nerve paralysis, and generalized cerebral atrophy/hypoplasia, among others.

Terms associated with poor emotional regulation and abnormalities in the amygdala, putamen, or cerebellum size include apathy, stereotypy, hallucinations, self-injurious behavior, generalized cerebral atrophy/hypoplasia, progressive global developmental delay, cranial nerve paralysis, and emotional dysregulation. This enrichment in both pathways and symptoms ratify the role that these regions play not only in brain morphology and development, but also in the appearance of clinical symptoms associated with NDDs.

In summary, our analyses in the large UKBB database indicate that NDD-risk regions affect brain morphology, and that some characteristic features of these neuroanatomical abnormalities are associated with the increased likelihood of risk for NDDs and other neuropsychiatric disorders. Although the sample size is a limiting factor, affecting the statistical power to detect significant differences between individuals with and without NDDs, our findings still reveal morphological characteristics that could potentially drive risk, such as altered cortical thickness and putamen enlargement, which may serve as intermediate phenotypes linking genetic variants to clinical outcomes. Furthermore, we reported non-clinical penetrance estimates, and we were able to ratify the risk of the morphological abnormalities associated with specific regions, since the genes contained in them were significantly implicated in both pathways and the clinical symptoms were frequently seen in NDDs and other neuropsychiatric disorders. These analyses allow us to broaden the range of risk factors that contribute to these diseases and to generate a catalog of both genetic risk variants and clinical symptoms that will allow for better diagnosis and treatment options for patients with these diseases.

## 4. Materials and Methods

### 4.1. UK Biobank Data and Cohort Selection

The UKBB is a large-scale biomedical database and research resource that includes data from over 500,000 participants aged 40–69 years, recruited between 2006 and 2010. It provides extensive phenotypic, imaging, and genomic data, making it a valuable tool for health-related research.

A cohort of psychiatric cases was created by selecting participants who either (i) ticked one or more options from the data field 20544 (mental health problems ever diagnosed by a professional), excluding the “Prefer not to answer” option (Appendix A.2) or (ii) were diagnosed with any disease under “Chapter V—Mental and behavioral disorders” according to the ICD10 classification in data fields 41270, 41202, and 41204 (Appendix A.3). It is important to note that data field 20544 contains self-reported diagnoses that can introduce some degree of misclassification. This limitation is further evaluated in Appendix A.4. To avoid association signals from neurodegenerative conditions affecting the structure and shape of the brain, we also removed patients with disorders such as Parkinson’s or Alzheimer’s disease (all excluded pathologies are detailed in Appendix A.4).

After applying these filters, only the participants with available brain morphological data were retained, with 9,798 participants being finally included in the psychiatric cohort.

On the other hand, individuals that do not meet any of the above criteria, i.e., no mental health problems or ICD10 code, were selected as controls. This resulted in a cohort of 22,131 control participants. Altogether, the total number of patients (i.e., cases and controls) considered for the analysis after the phenotype filtering was 31,929 (Figure 5).

### 4.2. MRI Data Analysis

Sixteen different MRI measures, i.e., image-derived phenotypes (IDPs), were obtained from all the participants from the FreeSurfer v6.0.0 (Charlestown, MA, USA) standard “aseg.stats” and “l/rh.aparc.stats” output files. These standard output files were previously generated by the UK Biobank Imaging team [58,59] from T1 sMRI DICOM files. More detailed information on the acquisition protocols, image processing pipeline, imaging data files, and IDPs is available in the UK Biobank Imaging Protocols. Brain areas in which this analysis focuses include cortical thickness (the average of the left and right hemispheres) as well as volumes of the amygdala, lateral ventricles, cerebellum, corpus callosum, pallidum, putamen, caudate, and hippocampus, all calculated by summing their left and right volumes. Other metrics that are taken from the output are total cerebral white matter, total gray matter, subcortical gray matter, cerebrospinal fluid, surface area, brain stem volume, and estimated total intracranial volume.

After acquiring the raw measures for the different structures, we standardized the values by calculating the Z-score of each IDP, considering the mean and standard deviation by sex and age group (including both cases and controls). Subsequently, we categorized the anatomical measure—also known as IDPs—into three different tiers: (i) IDP corresponding to a Z-score below −2, (ii) IDP corresponding to a Z-score above +2 standard deviations, and (iii) the measures with Z-scores between −2 and +2. In this sense, the abnormality of the neuroanatomical measure is defined when the IDP entails Z-score < −2 or Z-score > 2, which identifies relative changes in brain structures across groups.

### 4.3. Enrichment Analysis of Morphological Traits in Cases vs. Controls

After that, we determined whether these aberrant morphological traits (AMTs), i.e., IDPs showing both Z-score < −2 or Z-score > 2, are more prevalent in cases with psychiatric diagnoses compared to controls. Therefore, we performed a Fisher’s Exact Test (contingency table: Table A2) for each IDP, to compare the proportions of individuals with AMTs between cases and controls. We also computed the prevalences of AMTs in cases and controls. We then performed a Wilcoxon exact test to evaluate whether there is a significant difference in the prevalence of AMTs between the two groups (e.g., cases vs. controls), testing the hypothesis that the prevalence is higher in cases compared to the controls.

The exactRankTests library was used since it handles ties (i.e., duplicated values) and provides exact *p*-values, which is crucial for non-normally distributed data with tied ranks.

### 4.4. Quality Control and Filtering of Genetic Data

CNV calls (generated using the MANTA v1.6 (San Diego, CA, USA) calling algorithm) from the UKBB selected participants were obtained from Halldorsson et al. [60]. We applied a series of filters to enhance the quality and specificity of the variants identified for subsequent analysis, as described below.

First, variants were required to have more than 25% of the reads supporting the variant (also known as alternative) allele. This threshold ensures that the alternative allele is supported by a significant proportion of the reads, implying reliability in the variant call. Additionally, a minimum depth of 10 reads was established for each variant to filter out low confidence calls that may arise from insufficient sequencing coverage.

In addition, only variants marked with a “PASS” in the FILTER column were retained. This filter includes variants that have passed the quality control checks inherent to the MANTA variant calling process. We also removed variants that fell into complex regions that are difficult to call. To achieve this, we discarded calls that overlapped 80% or more with these complex regions, with the percentage overlap being defined as the number of base pairs within the CNV region overlapping with complex regions divided by the length (i.e., the total number of base pairs) of the variant. The catalog of these complex regions can be found in the benchmarked genome stratifications from the Genome in a Bottle (GIAB) initiative (https://ftp-trace.ncbi.nlm.nih.gov/ReferenceSamples/giab/release/genome-stratifications/v3.4/GRCh38@all/Union/ (accessed on 19 December 2024)).

To further refine the dataset, only rare variants were retained, i.e., we discarded deletions (DELs) or duplications (DUPs) with at least 50% overlap with common variants (AF ≥ 1%) from the non-neuro gnomAD database. The overlap fraction is defined as the number of base pairs within the CNV region overlapping with common variants, divided by the total length of the CNV.

Finally, variants were annotated with their genomic region. These genomic regions were kept for the analysis only if they had an overlap of at least 10% with an NDD-risk region (Figure 4). NDD-risk regions were defined and derived from our previous work [25] comprising a total of 41 high confidence NDD-risk CNV loci. The full list of NDD-risk regions can be found in Supplementary Table S1. Also, in this case, as in the previously applied quality filters, the percentage of overlap is defined as the number of base pairs within the CNV region overlapping with an NDD-risk region, divided by the number of base pairs in the variant.

### 4.5. Enrichment Analysis of Variants in Psychiatric Cases vs. Controls

To assess whether carrying a specific genetic variant (deletion or duplication) in a risk region is significantly present in patients compared to controls, we performed a two-sided Fisher’s exact test (contingency table: Table A3).

### 4.6. Enrichment Analysis of Variants in Subjects with Abnormal Brain Morphological Traits

The objective was to assess whether individuals that carry certain genetic variants (deletions or duplications) in NDD-risk regions are more likely to present AMTs compared to individuals without the variants. Thus, we performed a two-sided Fisher’s exact test (contingency table: Table A4) to evaluate the association between the presence of variants and the abnormal brain morphological traits.

For each morphological trait analyzed (total brain volume, surface area, mean thickness, etc.), we divided subjects into two groups: those with an AMT—either with a Z-score less than −2 or Z-score greater than 2 for that specific feature—and those who did not present any aberrant trait. The presence or absence of variants (deletions or duplications) in NDD-risk regions was then analyzed for both groups. In this way, we evaluated aberrant traits above the norm (Z-score > 2) and below the norm (Z-score < −2) for all the 16 morphological measures for the 24 NDD-risk regions assessed either on deletion or duplication, resulting in 1023 tests.

### 4.7. Adjustment for Correlated Measures of Brain Regions

**Figure A1:**
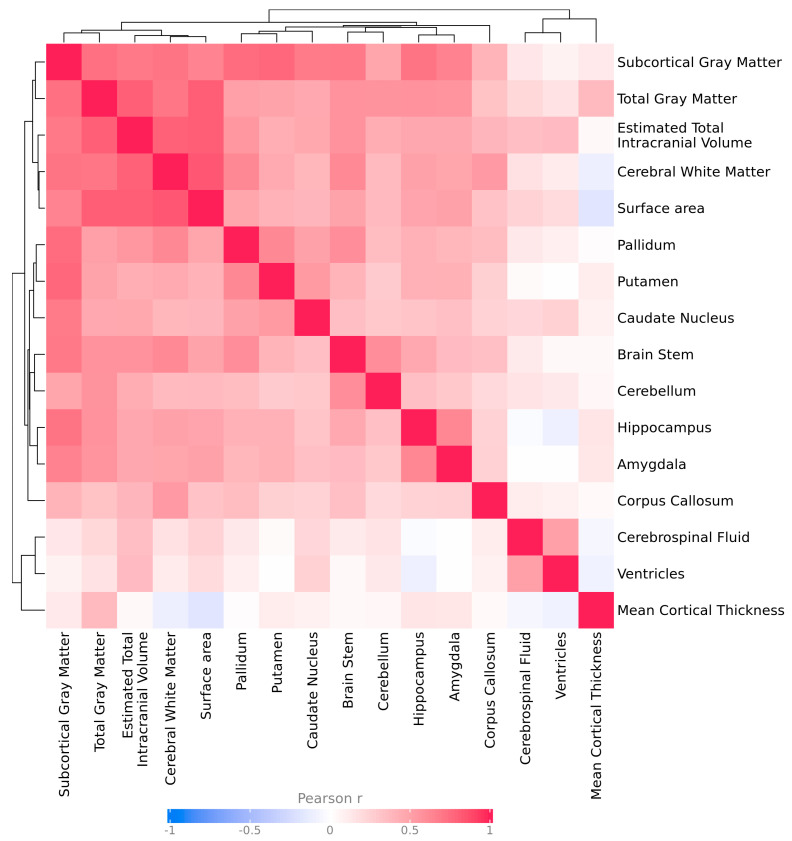
The heatmap of pairwise Pearson correlations. The heatmap displays the Pearson correlation coefficients among the different brain measures. Colors represent the strength and direction of correlations, from negative (blue) to positive (red). Rows and columns are clustered to highlight patterns of similarity. The correlation coefficient legend is shown below the heatmap for reference.

To account for correlations between brain region volumes and avoid overly conservative multiple test correction thresholds, we estimated the effective number of independent brain region tests (M_eff_). The standard Bonferroni correction assumes that all tests are independent, which is not the case for brain morphometric features, which are often correlated due to shared biological pathways or anatomical proximity; see Figure A1.

We calculated M_eff_ using the Galwey method, which establishes the effective number of independent variables from the eigenvalues of the correlation matrix between all brain regions included in the analysis. In our case, we performed the correlation matrix with Pearson’s Z-score values of the different variables for each patient. In this way, an eigen decomposition of the correlation matrix is performed and the variance explained by each principal component is summed to quantify how many independent dimensions (i.e., truly non-redundant tests) remain after taking intercorrelations into account.

In our data, the correlation structure showed a cluster of strongly correlated brain morphological traits, resulting in an estimated M_eff_ of 10 independent brain morphological traits (out of a total of 16). Combining this with the 33 tested CNVs, we applied an adjusted Bonferroni threshold of 0.05 divided by (10 × 33), resulting in a significance threshold of α = 1.5 × 10^−4^.

This approach provides more appropriate control for multiple testing when traits are not fully independent, ensuring that we balance type I error control with the risk of discarding biologically relevant signals.

### 4.8. Calculation of Prevalence of AMT in UKBB Subjects

To assess the prevalence of the abnormalities in brain morphology in the UKBB population, we calculated the number of individuals with an AMT compared to the total population by IDP. We also calculated the prevalence by cases and controls (the number of cases or controls with an AMT divided by the total number of cases or controls). Finally, we computed the prevalence of an AMT by CNVs, i.e., the number of carriers of a specific deletion or duplication with either a Z-score < −2 or Z-score > 2, compared to the total number of carriers of the variant.

### 4.9. Gene Set Enrichment Analysis and Functional Interpretation of Genes in NDD Risk Regions

We performed a gene set enrichment analysis to assess the biological implications of the genes located within CNV regions nominally significantly associated with aberrant brain morphology (i.e., genomic regions derived from the analysis that show *p*-value < 0.05 for AMTs in CNV carriers). We performed a Gene Ontology (GO) enrichment analysis using R 4.4.2 and the libraries org.Hs.eg.db_3.20.0 and clusterProfiler_4.14.3. We used an FDR < 0.05 threshold for multiple test correction to avoid false positive signals. We also performed the same exact test on the subset of dosage-sensitive genes located within the CNVs regions associated with AMTs. To assess the interaction of the genes in the NDD-risk regions, we performed a PPI network analysis using the STRINGdb R package (version 9.05), establishing the threshold level of interaction at 400.

Similarly, we also performed an enrichment of Human Phenotype Ontology (HPO) terms, i.e., we evaluated the HPO terms in our set of genes that were overrepresented. The genes employed for this analysis were both the full set of genes and the subset of dosage-sensitive genes within genomic regions that showed significant enrichment for AMTs in CNV carriers. We performed this analysis by using a published Shiny R tool called Phenoexam [61], using an FDR threshold < 0.05 to correct for multiple testing.

To assess the enrichment of known NDD-associated phenotypes in the Human Phenotype Ontology (HPO) terms enriched in our gene set, we first curated a list of NDD-related HPO terms from an Orphanet-derived catalog [33], selecting terms linked to developmental conditions. We performed a Fisher’s Exact Test, and it was conducted both on the uncorrected and corrected significantly enriched HPO terms to determine whether NDD-related phenotypes were statistically overrepresented in the gene set. Lastly, we annotated the significantly enriched terms that overlapped with the NDD catalog as “Previously identified NDD phenotype/clinical symptom” (Supplementary Table S9).

To robustly assess the statistical significance of our HPO enrichment results, we employed an approach based on gene set permutations. Specifically, we generated 200 random gene sets of the same number of genes than those in the regions that were significantly associated with an AMT (n = 464 for the full test/n = 91 for the subset of dosage-sensitive genes). For each randomly selected gene set, we performed the same HPO enrichment analysis as with the original data. We then obtained an empirical *p*-value by calculating the frequency over 200 permutations in which a term was equally or more enriched (i.e., same or smaller *p*-value) than in the observed analysis (i.e., the number of tests with a *p*-value < or = the observed *p*-value/200 tests). In addition, the proportion of qualifying genes—those located in NDD-risk regions associated with AMTs—that are linked to each NDD-related phenotype (the observed gene overlap ratio) was compared to the average proportion of genes from the 200 permutations linked to the evaluated HPO terms (i.e., gene-overlap ratios expected by chance).

## Data Availability

The original contributions presented in this study are included in the article and Supplementary Materials. Further inquiries can be directed to the corresponding author.

## Supplementary Materials

The following supporting information can be downloaded at: https://www.mdpi.com/article/10.3390/ijms26157062/s1.

## Abbreviations

TIV	Total Intracranial Volume
SA	Surface Area
CT	Cortical Thickness
CNV	Copy Number Variant
AMT	Aberrant Morphological Trait
NDD	Neurodevelopmental Disorder
UKBB	UK Biobank
GO	Gene Ontology
HPO	Human Phenotype Ontology
EEG	Electroencephalography
ICD10	International Classification of Diseases, 10th Revision
MRI	Magnetic Resonance Imaging
IDP	Imaging-Derived Phenotype
FDR	False Discovery Rate
Kb	Kilo Bases, Measure Representing 1000 Base Pairs
Mb	Mega Bases, Measure Representing 1,000,000 Base Pairs

## Appendix A

### Appendix A.1. Power Calculation

To assess the statistical power of an unmatched case–control study investigating the association between a genetic variant and AMTs in neuropsychiatric disorders, we used standard epidemiological formulas. Power represents the likelihood of correctly identifying an association if one truly exists.

We used the following parameters to compute the scores:

- p0, the proportion of controls with the variant (p0 = 254/21,877);
- RR, relative risk, representing the increased risk of NDD abnormal morphology in exposed carrying cases relative to controls (RR = (117/9,681)/p0);
- Sample sizes cases (n = 9,798);
- Sample sizes controls (n = 22,131).

Using p0, we estimated the variances in exposure between cases and controls, which reflect variability due to sample size. The standardized effect size was then determined by dividing the difference in exposure probabilities by the combined variance across groups, allowing us to gauge how pronounced the effect is, adjusted by the study size.

Finally, we used the effect size to determine power against a two-sided Z-test with a significance level of 0.05.

**Table A1:** Summary table for the power calculation.

Type of “Exposure”	Type of Cohort	No. of Unique Patients
No variant	case	9,681
No variant	control	21,877
Variant	case	117
Variant	control	254

### Appendix A.2. Mental Health Problems Ever Diagnosed by a Professional (Data Field 20544)

The survey of mental health problems ever diagnosed by a professional was present in the UK Biobank data field 20544.

“Have you been diagnosed with one or more of the following mental health problems by a professional, even if you do not have it currently? (Tick all that apply):” (a participant was offered the following set of options):

- Social anxiety or social phobia;
- Schizophrenia;
- Any other type of psychosis or psychotic illness;
- A personality disorder;
- Any other phobia (e.g., disabling fear of heights or spiders);
- Panic attacks;
- Obsessive–compulsive disorder (OCD);
- Mania, hypomania, bipolar, or manic depression;
- Depression;
- Bulimia nervosa;
- Psychological over-eating or binge-eating;
- Autism, Asperger’s, or autistic spectrum disorder;
- Anxiety, nerves, or generalized anxiety disorder;
- Anorexia nervosa;
- Agoraphobia;
- Attention deficit or attention deficit and hyperactivity disorder (ADD/ADHD);
- Prefer not to answer.

### Appendix A.3. Diagnoses—ICD10 (Data Fields 41270, 41202, and 41204)

Diagnoses included in the analysis. Patient having one or more diagnosis in this list were considered for the study of association between abnormal brain morphological features and the presence of NDD-risk CNVs.

- Social anxiety or social phobia:
  ◦ F40.1 → social phobias.
  ◦ F93.2 → social anxiety disorder of childhood.
- Schizophrenia:
  ◦ F20 → schizophrenia.
- Any other type of psychosis or psychotic illness:
  ◦ F29 → unspecified nonorganic psychosis.
  ◦ F10.7 → residual and late-onset psychotic disorder.
  ◦ F10.5 → mental and behavioral disorders due to use of alcohol.
  ◦ F11.5 → mental and behavioral disorders due to use of opioids.
  ◦ F12.5 → mental and behavioral disorders due to use of cannabinoids.
  ◦ F13.5 → mental and behavioral disorders due to use of sedatives or hypnotics.
  ◦ F14.5 → mental and behavioral disorders due to use of cocaine.
  ◦ F15.5 → mental and behavioral disorders due to use of other stimulants, including caffeine.
  ◦ F16.5 → mental and behavioral disorders due to use of hallucinogens.
  ◦ F17.5 → mental and behavioral disorders due to use of tobacco.
  ◦ F18.5 → mental and behavioral disorders due to use of volatile solvents.
  ◦ F19.5 → mental and behavioral disorders due to multiple drug use and use of other psychoactive substances.
  ◦ F23 → acute and transient psychotic disorders.
  ◦ F28 → other nonorganic psychotic disorders.
  ◦ A personality disorder.
  ◦ F60 → specific personality disorders.
  ◦ F61 → mixed and other personality disorders.
  ◦ F62 → enduring personality changes, not attributable to brain damage and disease.
  ◦ F68 → other disorders of adult personality and behavior.
  ◦ F69 → unspecified disorder of adult personality and behavior.
  ◦ F07 → personality and behavioral disorders due to brain disease, damage, and dysfunction.
- Any other phobia (e.g., disabling fear of heights or spiders):
  ◦ F40.2 → specific (isolated) phobias.
  ◦ F40.8 → other phobic anxiety disorders.
  ◦ F40.9 → phobic anxiety disorder, unspecified.
- Panic attacks:
  ◦ F41.0 → panic disorder [episodic paroxysmal anxiety].
  ◦ F43 → reaction to severe stress and adjustment disorders.
- Obsessive–compulsive disorder (OCD):
  ◦ F42 → obsessive–compulsive disorder.
- Mania, hypomania, bipolar, or manic depression:
  ◦ F30 → manic episode.
  ◦ F31 → bipolar affective disorder.
- Other factors influencing mental health status:
  ◦ Z86.4 → personal history of psychoactive substance abuse.
  ◦ Z81.3 → family history of other psychoactive substance abuse.
  ◦ Z71.6 → tobacco abuse counselling.
  ◦ Z71.4 → alcohol abuse counselling and surveillance.

### Appendix A.4. Excluded Diagnoses (ICD10-Data Fields 41270, 41202, and 41204)

List of excluded diagnosis. Patients in the UK Biobank cohort holding one or more of these diagnoses were excluded from the analyses to avoid biases or confounding factors due to other brain disorders that are not strictly neurodevelopmental.

- Dementia:
  ◦ F01 → vascular dementia.
  ◦ F02 → dementia in other diseases classified elsewhere.
  ◦ F03 → unspecified dementia.
  ◦ Alzheimer’s.
  ◦ G30 → Alzheimer’s disease.
  ◦ F00 → dementia in Alzheimer’s disease.
- Parkinson’s:
  ◦ G20 → Parkinson’s disease.
  ◦ G21 → secondary parkinsonism.
  ◦ G22 → Parkinsonism in diseases classified elsewhere.
  ◦ A52.1 → syphilitic parkinsonism.
- Others:
  ◦ B22.0 → HIV disease resulting in encephalopathy (HIV dementia).
  ◦ F05.1 → delirium superimposed on dementia.
  ◦ G31 → other degenerative diseases of nervous system, not elsewhere classified (Senile degeneration of the brain, degeneration due to alcohol, etc.).
  ◦ G93.4 → encephalopathy, unspecified.
  ◦ G10 → Huntington disease.
  ◦ C70.0→ malignant neoplasm of cerebral meninges.
  ◦ C71 → malignant neoplasm of brain.
  ◦ C72.8 → overlapping lesion of brain and other parts of central nervous system.
  ◦ C79.3 → secondary malignant neoplasm of brain and cerebral meninges.
  ◦ D33.0 → benign neoplasm of brain, supratentorial.
  ◦ D33.1 → benign neoplasm of brain, infratentorial.
  ◦ D33.2 → benign neoplasm of brain, unspecified.
  ◦ D43.0 → neoplasm of uncertain or unknown behavior of brain, supratentorial.
  ◦ D43.1 → neoplasm of uncertain or unknown behavior of brain, infratentorial.
  ◦ D43.2 → neoplasm of uncertain or unknown behavior of brain, unspecified.
  ◦ D32.0 → benign neoplasm of cerebral meninges.
  ◦ D42.0 → neoplasm of uncertain or unknown behavior of cerebral meninges.
  ◦ S06 → intracranial injury.

### Appendix A.5. Limitations in the Psychiatric Inclusion Criteria

One limitation of this study is that the classification of psychiatric status partially relies on self-reported data, which can introduce some degree of misclassification. For example, field 20544 captures self-reported diagnoses, which are generally considered less accurate than clinician-verified records. In contrast, fields 41270, 41202, and 41204 contain standardized ICD-10 diagnoses documented by clinicians. Also, previous research has demonstrated good concordance between self-reported and medical record diagnoses for some severe psychiatric conditions such as schizophrenia, with a positive predictive value of 0.8.

We acknowledge that self-reported questionnaires are not the most accurate tools for disease diagnosis. However, the UK Biobank dataset offers unique advantages in terms of sample size, the availability of high-quality imaging and genetic data, and extensive phenotypic information, and can inform on the penetrance and frequency of genetic variants in the general population—insights that would not be feasible to obtain from smaller, clinically ascertained samples. Therefore, despite these limitations, the UK Biobank remains a cornerstone resource for large-scale population studies like the present study.

## Appendix B

This appendix shows the contingency tables used in the assessment of the several enrichment analyses performed in this study.

**Table A2:** The contingency table illustrating the presence or absence of extreme morphological traits (AMTs), defined as Z-scores < −2 or >2, among individuals with psychiatric diagnoses compared to controls.

	Cases	Controls
AMT Present	E	F
AMT Absent	G	H
Total (31,929 subjects)	E + G	F + H

**Table A3:** The enrichment of genetic variants in psychiatric cases versus controls: the contingency table showing the distribution of individuals carrying or not carrying a genetic variant within known risk regions among psychiatric cases and controls.

	Cases	Controls
Carrier	I	J
Non-carrier	K	L
Total (31,929 subjects)	I + K	J + L

**Table A4:** The contingency table for abnormal brain morphological traits and the presence of a qualifying variant within a specific NDD-risk region. The distribution of variant carriers and non-carriers among subjects with and without extreme brain morphological traits (Z < −2 or Z > 2).

	Subjects with AMT (Z-Score < −2 or Z-Score > 2)	Subjects Without AMT
Variant Present	A	B
No Variant Present	C	D
Total (31,929 subjects)	A + C	B + D
